# Drift and Directional Selection Are the Evolutionary Forces Driving Gene Expression Divergence in Eye and Brain Tissue of *Heliconius* Butterflies

**DOI:** 10.1534/genetics.119.302493

**Published:** 2019-08-29

**Authors:** Ana Catalán, Adriana D. Briscoe, Sebastian Höhna

**Affiliations:** * Department of Evolutionary Biology, Evolutionary Biology Centre (EBC), Uppsala University, 75236, Sweden; † Division of Evolutionary Biology, Ludwig-Maximilians-Universität München, Planegg-Martinsried 82152, Germany; ‡ Department of Ecology and Evolutionary Biology, University of California, Irvine, California 92697; § Department of Earth and Environmental Sciences, Paleontology and Geobiology, 80333 Munich, Germany; ** GeoBio-Center, Ludwig-Maximilians-Universität München, 80333 Munich, Germany

**Keywords:** Brownian motion, natural selection, stabilizing selection, Ornstein–Uhlenbeck, RevBayes

## Abstract

Investigating gene expression evolution over micro- and macroevolutionary timescales will expand our understanding of the role of gene expression in adaptation and speciation. In this study, we characterized the evolutionary forces acting on gene expression levels in eye and brain tissue of five *Heliconius* butterflies with divergence times of ∼5–12 MYA. We developed and applied Brownian motion (BM) and Ornstein–Uhlenbeck (OU) models to identify genes whose expression levels are evolving through drift, stabilizing selection, or a lineage-specific shift. We found that 81% of the genes evolve under genetic drift. When testing for branch-specific shifts in gene expression, we detected 368 (16%) shift events. Genes showing a shift toward upregulation have significantly lower gene expression variance than those genes showing a shift leading toward downregulation. We hypothesize that directional selection is acting in shifts causing upregulation, since transcription is costly. We further uncovered through simulations that parameter estimation of OU models is biased when using small phylogenies and only becomes reliable with phylogenies having ≥ 50 taxa. Therefore, we developed a new statistical test based on BM to identify highly conserved genes (*i.e.*, evolving under strong stabilizing selection), which comprised 3% of the orthoclusters. In conclusion, we found that drift is the dominant evolutionary force driving gene expression evolution in eye and brain tissue in *Heliconius*. Nevertheless, the higher proportion of genes evolving under directional than under stabilizing selection might reflect species-specific selective pressures on vision and the brain that are necessary to fulfill species-specific requirements.

## Abstract (precis)

Characterization of gene expression patterns across species - and the evolutionary forces driving them - can reveal processes that have remained conserved across species, as well as those that have changed in a species- specific manner...

Species and populations diverge through the accumulation of genetic changes that affect coding or non-coding genomic regions that Genetic variation affecting gene expression has the potential of changing gene expression patterns in a spatiotemporal manner by changing gene expression profiles in specific organs and cell types at particular developmental stages (Carroll 2005; Signor and Nuzhdin 2018). This spatiotemporal attribute of gene expression might enable evolutionary change in a compartmentalized manner, allowing for change where it is required but also allowing for the needed processes to remain conserved. Phenotypic diversity caused by changes in gene expression encompasses a great variety of traits, including changes affecting an organism’s coloration (Nadeau 2016), size, and shape (Ahi *et al.* 2017), as well as sensory perception and behavior, among other phenotypes (Lee *et al.* 2000; Wanner *et al.* 2007). Even though major advances have been made in linking gene expression variation to a phenotype (Catalán *et al.* 2016; Glaser-Schmitt and Parsch 2018), discerning the evolutionary forces that shape gene expression level variation among closely related species is an area that needs further research.

To understand the evolutionary forces acting on gene expression it is necessary to model within- and between-species gene expression variance. Neutral gene expression divergence between species leads to gene expression differences through divergence alone. Thus, neutral changes in gene expression provide a null hypothesis to detect deviations from the expected neutral gene expression divergence. A linear relationship between divergence time and gene expression variance has been proposed for closely related species, assuming a clock-like (*i.e.*, constant through time) rate of gene expression divergence (Khaitovich *et al.* 2004, 2005a). Another approach to study the evolutionary forces shaping gene expression evolution, which is motivated by statistical phylogenetics, is fitting Brownian motion (BM) models. BM models are often used to describe the rate of change of continuous traits through time taking into account the known phylogeny of the taxa of interest (Felsenstein 1985). Thus, in a BM context, the parameter σ^2^ is often described as the volatility parameter that determines the rate at which a trait’s value diffuses away from its current state (Bedford and Hartl 2009). Fitting BM models to investigate gene expression evolution has shown that stabilizing selection and evolution through drift can be readily characterized (Kalinka *et al.* 2010; Wong *et al.* 2015).

Ornstein–Uhlenbeck (OU) models have also been used to study continuous trait evolution in a phylogenetic context (Hansen 1997; Butler and King 2004). OU models, an extension to BM models, include two extra parameters, α and θ. As in a BM context, σ^2^ is the rate at which a trait changes through time and the parameter α is the force pulling back the diffused trait to an optimum state. This is analogous to stabilizing selection pulling back a trait to its optimum value after having experienced a departure from it. θ is described as the trait’s optimum state at a particular time point toward which the pull of α is aimed (Hansen 1997; Butler and King 2004). OU models offer a useful framework to generate hypotheses about the evolutionary forces acting on transcriptome levels, whether it is drift, stabilizing selection, or directional selection (Bedford and Hartl 2009; Rohlfs and Nielsen 2015; Wong *et al.* 2015; Chen *et al.* 2017; Stern and Crandall 2018).

In this study, we used five closely related species of *Heliconius* butterflies to explore the evolutionary forces shaping gene expression variation in combined eye and brain tissue. *Heliconius charithonia*, *H. sara*, *H. erato*, *H. melpomene*, and *H. doris* (Figure 1) belong to four of the seven distinct *Heliconius* phylogenetic clades with divergence times ranging from 5.5 to 11.8 MYA. Beside showing great diversity in wing color patterns (Kronforst and Papa 2015), *Heliconius* butterflies also show diversity in life history traits (Salcedo 2010; Merrill *et al.* 2015), mating systems (Beltrán *et al.* 2007; Walters *et al.* 2012), and behavior (Mendoza-Cuenca and Macías-Ordóñez 2005; Merrill *et al.* 2019). Since *Heliconius* butterflies are diurnal species, visual stimuli provide key sources of information about the environment. For example, flowers and oviposition sites, potential mates, or predators are all targets of interest to butterflies in which the first line of perception is visual (Finkbeiner *et al.* 2014, 2017). After visual cues are detected by the visual system, the detected information travels to the brain where it is processed, and its output can result in a specific behavior or physiological response. Thus, the brain’s processing and output together with the visual system have the potential of being finely tuned according to a species’ life history. In the case of *Heliconius* butterflies, high diversity of adult compound eye retinal mosaics (between sexes and species) has been described (McCulloch *et al.* 2016, 2017), as well as species-specific differences in brain morphology (Montgomery *et al.* 2016). Which evolutionary forces are shaping adult eye and brain expression in *Heliconius* is one question we seek to investigate, and in that way, gain an understanding of the potential role of interspecies gene expression differences in speciation and adaptation.

**Figure 1:**
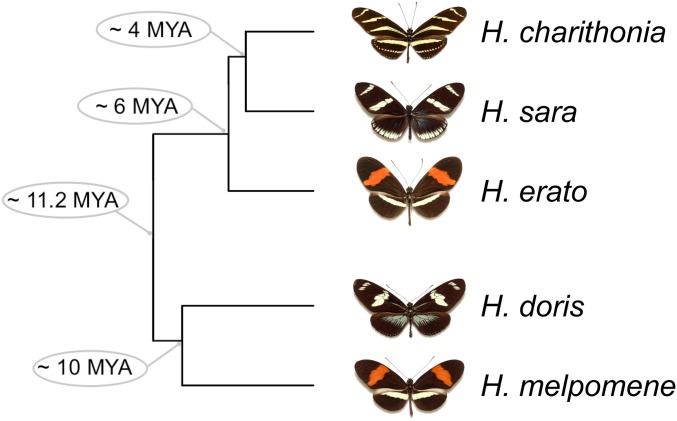
Phylogenetic relationship of the *Heliconius* species used in this study showing divergence times at each node (Kozak *et al.* 2015).

Therefore, in this study we investigated which evolutionary forces are driving gene expression variation in combined eye and brain tissue. More specifically, we aimed to identify if expression variation in individual genes is evolving, for example, through drift, stabilizing selection, or directional selection. To this end, we generated a set of orthoclusters shared among our five butterfly species together with expression data for each gene in each orthocluster. We characterized the selective forces acting on gene expression levels, thereby revealing the fraction of the transcriptome evolving under drift, directional selection, and stabilizing selection.

## Materials and Methods

### Data set, samples, and RNA-sequencing

*De novo* transcriptome assemblies for *H. charithonia*, *H. sara*, and *H. doris* were downloaded from Dryad with data identifier doi: 10.5061/dryad.ds21fv5 (Catalán *et al.* 2018). Transcriptomes from *H. erato* and *H. melpomene* were downloaded from Dryad with data identifier doi: 10.5061/dryad.8d724 (Smith *et al.* 2016). RNA-sequencing (RNA-seq) data for all the species were downloaded from ArrayExpress: E-MTAB-6810 (Catalán *et al.* 2018). The RNA-seq data were generated as follows: pupae were obtained from The Butterfly Farm, Costa Rica Entomological Supply and allowed to eclose under controlled laboratory conditions.

Biological replicates for females (F) and males (M) for each species were generated: *H. charithonia* (F = 6, M = 6), *H. sara* (F = 5, M = 6), *H. erato* (F = 3, M = 3), *H. doris* (F = 6, M = 6), and *H. melpomene* (F = 4, M = 4). Three days after eclosion, the butterflies were flash frozen at −80° until RNA extraction. Combined eye and brain tissue was dissected from the same individual by removing the antennae, palps, and proboscis from the head capsule. RNA was extracted using TRIzol (Thermo Fisher Scientific, Waltham, MA) following the manufacturer’s instructions. RNA was purified using a NucleoSpin RNA II kit (Macherey-Nagel, Bethlehem, PA). Purified RNA was quantified using a Qubit 2.0 Fluorometer (Thermo Fisher Scientific) and RNA integrity was checked using an Agilent Bioanalyzer 2100 (Agilent Technologies, Santa Clara, CA), with RNA integrity number (RIN) ranging from 8 to 10. RNA-seq libraries were prepared for each individual using a TruSeq RNA Sample Preparation Kit v2 (Illumina, San Diego, CA). Double-stranded cDNA libraries were quantified, quality checked, normalized, and pooled for sequencing according to their concentrations. Pooled libraries were run on a 2% agarose gel to size select fragments of ∼240–600 bp. cDNA was recovered using a Geneclean III kit (MP Biomedical, Santa Ana, CA) and purified using Agencourt AMPure XP beads (Beckman Coulter, Brea, CA). Sequencing was conducted at the University of California, Irvine Genomics High-Throughput Facility using a HiSeq 2500 (Illumina), paired end 100-cycle sequence run.

Reads were mapped to their corresponding transcriptome using Bowtie (Langmead *et al.* 2009), and FPKM (fragments per kilobase per million mapped read) values were calculated for each species and used for downstream analysis (Catalán *et al.* 2018). Annotation files for each transcriptome were downloaded from doi: 10.5061/ dryad.ds21fv5 (Catalán *et al.* 2018) with the exception of the transcriptome annotation for *H. erato*, which was newly annotated. For the annotation of the *H. erato* transcriptome, TransDecoder (version 5.0.2) was used to identify candidate coding regions. The predicted coding sequences were utilized to identify orthologous hits in the UniProt, FlyBase, and Pfam databases using blastp (2.2.30) (Altschul *et al.* 1990; Chintapalli *et al.* 2007; Punta *et al.* 2012) (Appendix I).

### Orthology assessment

Orthology across species was determined by using the Unrooted Phylogenetic Orthology (UPhO) pipeline (Ballesteros and Hormiga 2016). UPhO uses an all-species pairwise blastp search and a Markov clustering algorithm (MCL) (version 1.0.0) (Enright *et al.* 2002) to cluster sequences according to sequence similarity. Clustered sequences were aligned with MAFFT (version 7.3.05) (Katoh and Toh 2008) and curated after alignment with trimAl (version 1.3) (Capella-Gutiérrez *et al.* 2009). Phylogenetic inference for each sequence cluster was done using RAxML (version 8.2.10) (Stamatakis 2006) and orthology was assessed for each generated tree using the UPhO algorithm. A matrix with log_2_ FPKM values was generated for each orthocluster, which was used to analyze and model gene expression variance (Appendix I).

### Modeling gene expression evolution

To study the forces driving gene expression evolution, we implemented a set of six different statistical models (Table 1). Each one models the mean species gene expression level (between-species variance) and the gene expression levels of individual samples per species (within-species variance). How these mean species gene expression levels evolve, or not, along the phylogeny and over time, is specific and central to each model. We estimated the parameters of each model and performed Bayesian model selection using Bayes factors to establish which model describes the observed data best, and thus which process is most likely to drive gene expression evolution in the five *Heliconius* species of our study (see below).

**Table 1:** Summary of the models implemented in this work

Model	Description	Parameters
Equal species means	All species have the same mean gene expression level	μ: global mean gene expression level
Unequal species means	All species have their own independent mean gene expression level	μ_i_: mean gene expression level per species
Brownian motion	Random drift of the species’ mean gene expression level along the phylogeny	σ^2^: rate of drift
Brownian motion with shift	Random drift with one branch having a different rate (directional selection)	σ^2^_B_: rate of drift background branch
		σ^2^_F_: rate of drift foreground branch
Ornstein–Uhlenbeck	Stabilizing selection of the species mean gene expression level evolving along the phylogeny	σ^2^: rate of drift
		α: strength of selection
		θ: optimal gene expression level
Ornstein–Uhlenbeck with shift	Directional selection due to a shift in optimal gene expression level	σ^2^_B_: rate of drift background branch
		σ^2^_F_: rate of drift foreground branch.
		θ_B_: optimal gene expression level background branch
		θ_F_: optimal gene expression level foreground branch
		α: strength of selection

The simplest model of gene expression assumes that all species have the exact same mean gene expression level. In this case, we only have one parameter, μ, which defines the mean gene expression level of all species. The expression level X_ij_ of individual *i* from species *j* is modeled using a normal distribution with X_ij_ ∼ Norm(μ, δ^2^_i_). We chose a uniform prior distribution between −20 and +20 for the mean gene expression parameter μ. Note that we assume that every species has its own gene expression variance parameter δ^2^_i_ (see below). This model assumes that there is no evolution of gene expression levels, *i.e.*, gene expression levels are completely conserved among species.

The second model that we implemented was a model where each species has its own gene expression mean μ_i_. Thus, we modeled the gene expression level X_ij_ of gene *i* from species *j* using a normal distribution with X_ij_ ∼ Norm(μ_i_, δ^2^_i_). In this model, each species has a different mean gene expression level, but these gene expression levels do not evolve under an evolutionary model; they are intrinsically different without any mechanistic reason (no phylogenetic signal). As with the first model, we assumed a uniform prior distribution between −20 and +20 for each mean gene expression level μ_i_.

The third model that we implemented was a phylogenetic BM model (Felsenstein 1985). We assume that any gene expression value at the root of the phylogeny is equally probable. Then, the mean gene expression levels μ evolve along the lineages of the phylogeny. The BM model specifies that the focal variable, μ in our case, is drawn from a normal distribution centered around the value of the ancestor, μ_A_. The amount of change, *i.e.*, the rate of random drift, is defined by the parameter σ^2^. We assumed a log-uniform prior distribution between 10E−5 and 10E5 for the drift parameter σ^2^. Thus, the mean gene expression levels μ_i_ for the species of the phylogeny are distributed according to a multivariate normal distribution where the covariance structure is defined by the phylogeny (Felsenstein 1985). This means that more closely related species are expected to have a more similar mean gene expression level because they share more evolutionary history (*i.e.*, they are more recently diverged), which is modeled by the covariance structure. Such a phylogenetic model of gene expression evolution has been applied previously by Bedford and Hartl (2009). Importantly, the BM model only defines how the mean gene expression levels evolve but does not allow for any sample variance of the individuals of a species. Therefore, we extended the standard phylogenetic BM model to allow for within-species sample variance, where again the expression level X_ij_ of individual *i* from species *j* is normally distributed with X_ij_ ∼ Norm(μ_i_, δ^2^_i_) where δ^2^_i_ is the within-species variance parameter (Ives *et al.* 2007; Rohlfs and Nielsen 2015). This extension to allow for within-species variance was developed for all phylogenetic models (BM, BM with shift, OU, and OU with shift).

The fourth model that we implemented was a phylogenetic BM model with branch-specific rates of evolution, thus detecting directional selection. The mean gene expression level evolves under a BM model (*i.e.*, random drift) where the rate of evolution for branch *k* is given by σ^2^_k_ (O’Meara *et al.* 2006; Eastman *et al.* 2011). Thus, a branch with a higher rate of evolution σ^2^_k_ signifies more change in gene expression levels than under a constant rate random drift model (the BM model). Directional selection can therefore be detected by inferring an elevated estimate of σ^2^_k_ compared with the background rate of drift σ^2^. Specifically, we applied a background rate of drift σ^2^_B_ to all branches except the chosen foreground branch, which received its own rate of drift σ^2^_F_.

The fifth model we implemented was a phylogenetic OU process (Hansen 1997). Similar to BM, the OU process models the evolution of the mean gene expression level per species along a phylogenetic tree. However, unlike BM, the mean expression level diffuses with rate σ^2^ and is attracted with strength α to an optimum level θ. Thus, the OU process has an expected variance of σ^2^/2α that is independent of time, *i.e.*, does not increase with increasing time but instead stabilizes. The variance becomes small if either the strength of selection is large or the rate of drift is small. This is, in fact, a major problem for OU models, which cannot distinguish if attraction (or selection) is strong or diffusion is weak (Ho and Ané 2014; Cooper *et al.* 2016).

The sixth model we implemented was an OU process with a branch-specific shift in both the rate of drift σ^2^ and the optimum gene expression level θ (Rohlfs *et al.* 2014; Uyeda and Harmon 2014). Thus, this branch-specific OU model is analogous to the branch-specific BM model, allowing for directional selection in an OU framework. Specifically, we tested if there was significant support for the chosen foreground branch, which received its own rate of drift σ^2^_F_ and optimum θ_B_ to be different from the background rate of drift σ^2^_B_ and optimum θ_B_. We used the same prior distributions as before, and assumed that both parameters for the background and foreground branches are drawn from the identical prior distribution. This model has, in total, five free additional parameters along with the five nuisance parameters (the within-species variances). Thus, we expect that this model is more prone to be overparameterized for our data set with five species. Nevertheless, our Bayesian approach for parameter estimation and model selection integrates over parameter uncertainty, and penalizes extra parameters by integrating over the prior distribution.

### Parameter estimation and model selection

We estimated parameters for our different models in a Bayesian statistical framework. Thus, we approximated the posterior distribution of the model parameters using Markov chain Monte Carlo (MCMC) sampling (Metropolis *et al.* 1953; Hastings 1970). We ran a separate MCMC analysis for each model and gene, 2393 analyses per model. Every model parameter was updated twice per MCMC iteration where the order of parameter updates was chosen randomly. We applied the same settings of the MCMC algorithm for each model. First, a burn-in phase of the MCMC algorithm was run for 2000 iterations with auto-tuning every 100 iterations. Then, the actual MCMC simulation was run for 50,000 iterations with sampling 10 iterations, yielding 5000 samples from the posterior distribution (Höhna *et al.* 2017).

Model selection was performed using marginal likelihoods. Marginal likelihoods are the probability of the data for a specific model integrated over all possible parameter values. From the marginal likelihood we can then compute Bayes factors and model probabilities (*i.e.*, weights of a model being the true model generating the data given a set of candidate models). We approximated the marginal likelihoods using stepping-stone sampling (Fan *et al.* 2011). The stepping-stone algorithm implemented in RevBayes consisted of 128 MCMC runs, where each MCMC run had the likelihood function raised to the power of β computed by the quantiles of a β probability distribution (Höhna *et al.* 2017).

### Data availability

The five different models that we used in our study are implemented in Bayesian phylogenetic inference software RevBayes v1.0.8 (Höhna *et al.* 2016). For efficient computation, we implemented the restricted maximum likelihood algorithm for BM (Felsenstein 1985) and OU models (FitzJohn 2012; Freckleton 2012). The source code and compiled executables of RevBayes are available from https://github.com/revbayes/revbayes, and tutorials about the analyses are available from https://revbayes.github.io/tutorials/. Supplemental material available at FigShare: https://doi.org/10.25386/genetics.9736253.

## Results

One of our main objectives in this study was to detect the evolutionary forces acting on gene expression by identifying deviations of gene expression variation from the variation expected from phylogeny alone. With this aim, we used transcriptomic data from combined eye and brain tissue of five *Heliconius* species (*H. charithonia*, *H. sara*, *H. erato*, *H. doris*, and *H. melpomene*) (Figure 1). From orthologous genes present and expressed in the five species, we built an expression matrix using log_2_-transformed FPKM values, which formed a data set of 2373 orthologous expressed genes (Appendix I). We used this data set to investigate the evolutionary forces shaping gene expression variation across species.

### Testing for equality of within-species variance in gene expression levels

**Figure 2:**
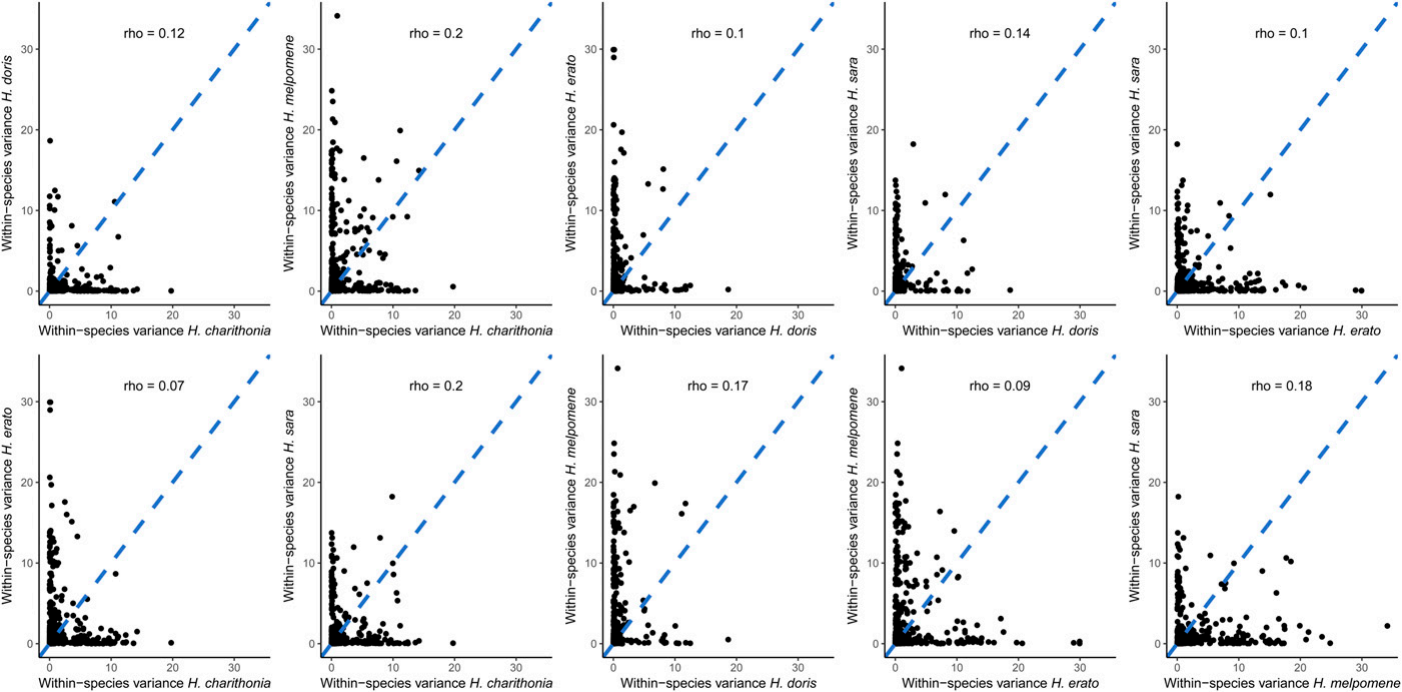
Pairwise correlation between five *Heliconius* species and their respective per gene expression variances. The correlation strength between per gene expression variances was estimated by calculating Pearson’s ρ correlation coefficient.

Previous models that assess the evolutionary forces acting on gene expression levels (Warnefors and Eyre-Walker 2012; Rohlfs *et al.* 2014) assume equal gene expression variance across species. Assuming equality of variance when it is not the case can lead to high type I error rates (Gastwirth *et al.* 2009). To test for equality of variance of gene expression across our five species, we calculated the within-species variance in gene expression. We calculated the within-species gene expression variance by calculating the gene expression variance from the mean, as measured in FKPMs, for each gene (as we have 6–12 biological replicates per species) and tested for a correlation of gene expression variance between every species pair (Figure 2).

In Figure 2, each black dot represents the value of the variance for each gene of the *Heliconius* species presented on either of the axes. From this pairwise assessment, we found no significant correlation among all possible pairs, with Pearsons’s ρ values ranging from 0.07 to 0.2 (Figure 2). Since gene expression variance across species is heterogeneous, and hence not correlated across species, we treated per gene expression variance as a random variable when fitting BM and OU models.

### Testing for a phylogenetic signal

**Figure 3:**
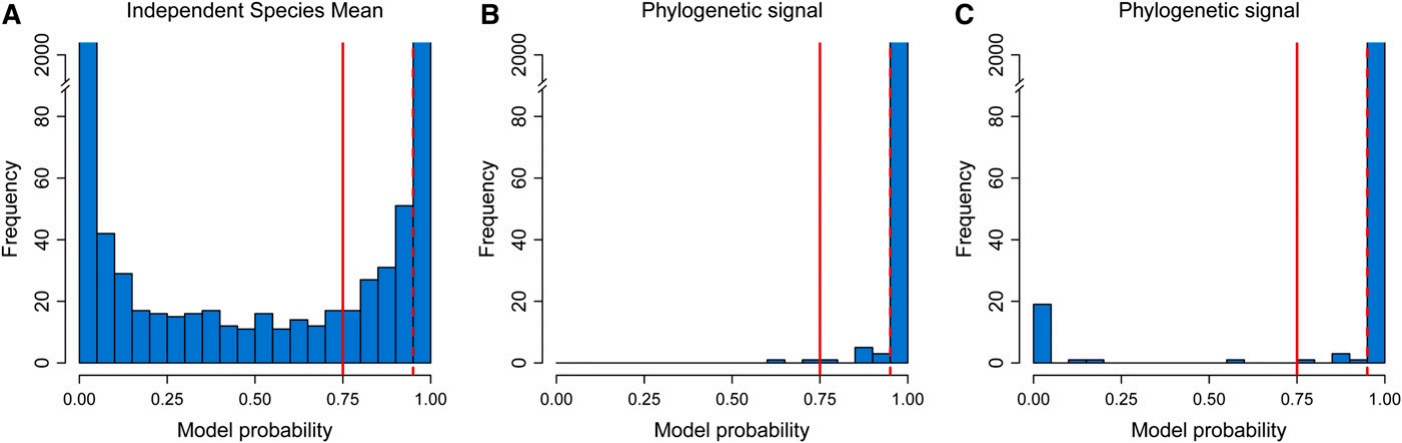
Testing for a phylogenetic signal in gene expression levels of *Heliconius* using BM. Significance is shown at model probability > 0.75 (solid red line, Bayes factor > 3, positive support) and model probability > 0.95 (dashed red line, Bayes factor > 20, strong support). (A) Shows the comparison between the two nonphylogenetic models (identical *vs.* independent species mean). (B) Shows the model probability of the BM model compared with the independent species mean model. (C) Shows the model probability of the BM model compared with the identical species mean model. BM, Brownian motion.

First, we started checking whether we could detect a phylogenetic signal from our gene expression data by using BM models. To test for this, we used two nonphylogenetic models where either all species had identical mean gene expression levels (model 1) or all species had their own independent mean gene expression levels (model 2). For each gene, we computed the probability of the BM model having produced the observed data, *i.e.*, a high probability means that it is more probable that the gene expression levels evolved under a BM model, whereas a low probability means that it is more probable that the gene expression levels evolved under a nonphylogenetic model (model 1 and model 2). A model probability of >0.75 corresponds to a Bayes factor of >3 (positive support) and a model probability of >0.95 corresponds to a Bayes factor of >20 (strong support) (Figure 3).

Our results show that for the majority of gene expression levels (2369 out of 2393) a phylogenetic signal can be recovered. Since RNA-seq data have high sensitivity to experimental and environmental noise, gene expression levels are prone to strong stochastic changes. The identification of a phylogenetic signal in most genes shows that BM models are suited to the investigation of the evolutionary forces acting on our gene expression data set.

### Testing for conserved gene expression levels

**Figure 4:**
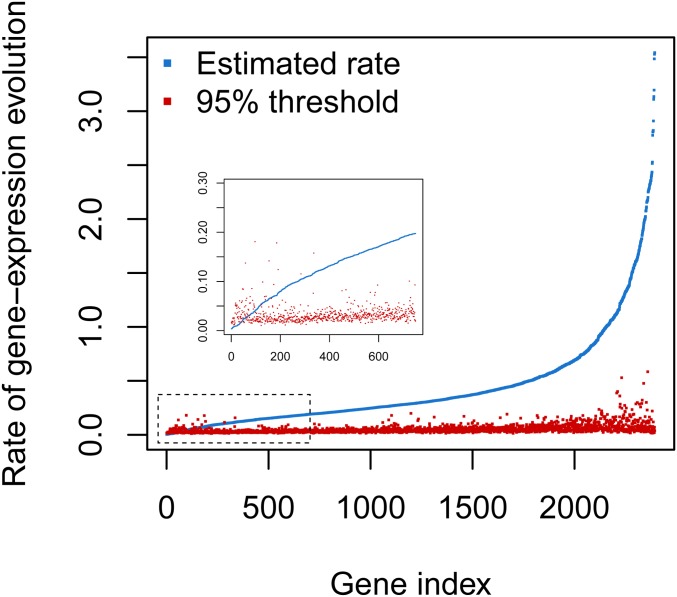
Posterior mean estimates of the rate of gene expression change (σ^2^ in blue) and the 95% threshold computed (red) using Monte Carlo simulations. The genes were sorted by an ascending estimate of σ^2^. Inset: close-up of genes whose σ^2^ is not significantly bigger than zero.

The next question we explored was how prevalent conserved gene expression levels are in combined eye and brain tissue of *Heliconius*. This question could be answered with our previous results by computing how often model 1, with identical species means, was recovered (Figure 2C). However, our model selection procedure relied on computing marginal likelihoods, which are intrinsically sensitive to the choice of prior distribution (Berger 1990; Kass and Raftery 1995; Sinharay and Stern 2002). Therefore, we additionally performed a sensitivity analysis of σ^2^ = 0 using Monte Carlo simulation as follows (Goldman 1993). First, we estimated the posterior distribution of all parameters under the identical species mean model (the only parameters were the per gene expression variances), then we used 1000 parameter samples from the posterior distribution to simulate gene expression data sets (*e.g.*, a data set consisting of a single gene with five species and 6–12 individuals per species) under the identical species mean model, yielding 1000 simulated data sets per gene in total. Then, for every gene of the 2393 genes, we estimated σ^2^ for each simulated data set as well as the original data set, which amounted to a total of 2,395,393 MCMC analyses. Finally, we calculated if the mean posterior estimate of the empirical data set was >95% of the mean posterior estimates of the simulated data sets. In cases where the mean posterior estimate of σ^2^ was not larger than the mean estimate of 95% of the simulated data sets, we concluded that these genes are highly conserved (Figure 4).

By using the described approach, we uncovered a set of 83 orthoclusters whose rates of gene expression evolution (Figure 4) and gene expression levels (Supplemental Material, Figure S1) across species are highly conserved. A σ^2^ not significantly different from zero can be caused by stabilizing selection hindering gene expression divergence, resulting in more similar gene expression patterns across different *Heliconius* species than expected.

### Testing for stabilizing selection acting on gene expression levels

Subsequently, we moved forward into implementing an OU to investigate the strength of stabilizing selection (Bedford and Hartl 2009; Beaulieu *et al.* 2012; Rohlfs *et al.* 2014). OU models include two extra parameters, α and θ. In a BM context, if σ^2^ is the rate at which a trait changes through time, α is then described as a force pulling back the diffused trait to an optimum state (θ).

**Figure 5:**
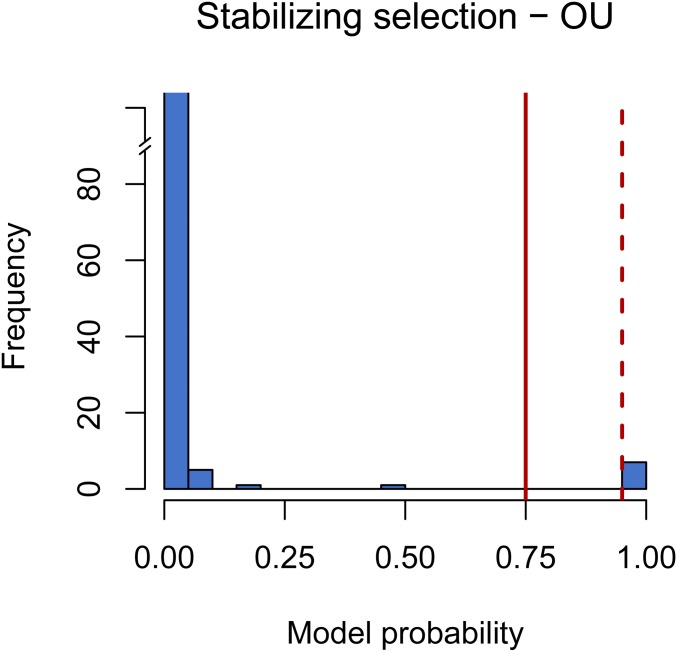
Model probability when testing model suitability when fitting an OU model for the assessment of stabilizing selection. Significance is shown at model probability > 0.75 (solid red, Bayes factor > 3, positive support) and model probability > 0.95 (solid red, Bayes factor > 20, strong support). There are only seven genes with significant support for stabilizing selection through an OU model. OU, Ornstein–Uhlenbeck.

We estimated the marginal likelihood for each gene under a BM model and an OU model. Then, we computed the probability (*i.e.*, support) of an OU model over a BM model using the marginal likelihoods. Our results show very low support for stabilizing selection under an OU model (Figure 5). When the marginal likelihoods were examined, in 99.7% of the cases a BM model explained our expression data better than an OU model.

### Testing the power to estimate stabilizing selection

**Figure 6:**
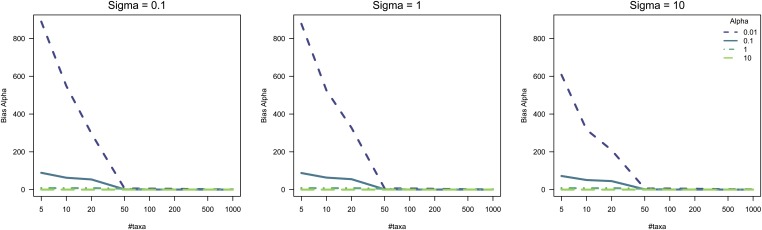
Simulation study for the assessment of parameter estimation bias under an OU model. The relative bias in estimates of the attraction/selection parameter (α) through 1000 simulations under σ values ranging from 0.1 to 10, and α values ranging from 0.01 to 10. Simulations were performed for phylogenies with sizes ranging from 5 to 1000 taxa. OU, Ornstein–Uhlenbeck.

Our results indicate that very few genes in *Heliconius* combined eye and brain tissue are evolving under stabilizing selection. It has previously been discussed that when working with small phylogenies (<10 species) there is a lack of power for parameter estimation when using an OU model (Beaulieu *et al.* 2012; Rohlfs *et al.* 2014), but no simulation studies have been done. By simulating data under an OU model using phylogenies with varying numbers of taxa, we were able to show how parameter estimation is biased. The attraction parameter α could only be estimated closely to the true values used for the simulations when the phylogenies contained ≥50 taxa. Thus, we can observe that the bias observed for parameter estimation drops considerably when the number of taxa composing the phylogeny reaches 50 (Figure 6). This observation holds as well for the estimation of σ^2^ under a range of σ values (Figures S2 and S3). Our simulation study shows that attention needs be paid when applying OU models to assess gene expression evolution for phylogenies containing <50 taxa.

### Detection of branch-specific shifts in gene expression

**Figure 7:**
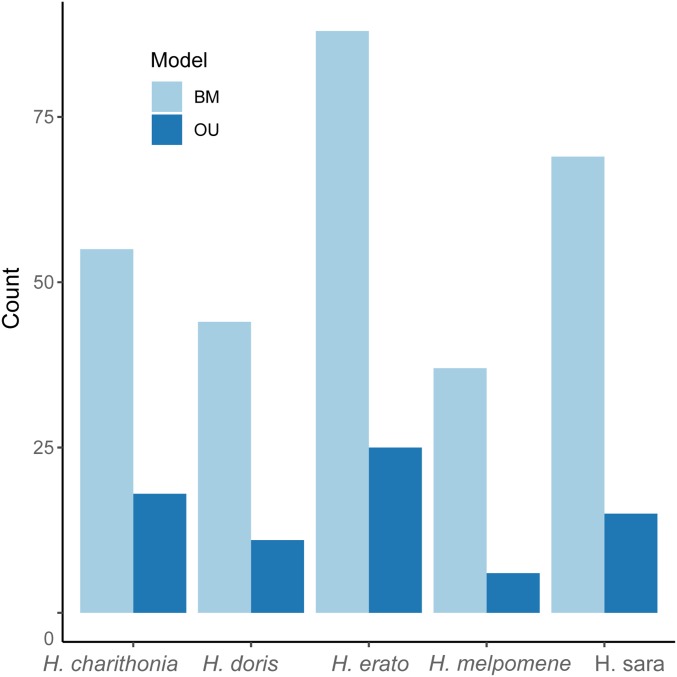
Bar plot showing branch-specific shifts on gene expression levels in *Heliconius*. Bars in light blue show branch shifts identified by BM models and dark blue bars show branch shifts identified by OU models. BM, Brownian motion; OU, Ornstein–Uhlenbeck.

To reveal genes whose gene expression patterns have putatively been shaped by directional selection, we tested for branch-specific shifts in evolutionary rates along the *Heliconius* tree. To explore branch-specific shifts in gene expression, we first used a BM model to test for the evolutionary rate (σ^2^) of a focal branch being different from the background rate (*i.e.*, the rest of the branches in the phylogenetic tree) and assessed significance by applying Bayes factors (Figure 7 and Figure S4). Second, we also tested branch-specific shifts through an OU model and tested for a branch-specific shift in gene expression level optimum (θ_F_) *vs.* the rest of the tree’s θ_B_.

With a BM approach, we were able to detect a total of 322 branch-specific shifts when considering only tip branches (Figure 7). We found 112 branch-specific shifts in the *H. erato* linage, 70 in *H. sara*, 67 in *H. charithonia*, 44 in *H. doris*, and 29 in *H. melpomene* (Figure 7 and Figure S5). *H. charithonia*, *H. sara*, and *H. doris* had more shifts toward a downregulation, although only in *H. charithonia* and *H. sara* was this difference significant (sign test, *H. charithonia: P*-value 6.738e−05 and *H. sara*: *P*-value 1.653e−06). In *H. erato* and *H. melpomene*, more upregulated genes were causing a branch-specific shift, although no significant difference was found.

When implementing an OU model, we recovered a total of 75 genes showing a branch-specific shift in gene expression optimum (Figure 7). From these genes, 55 also show a branch-specific shift when implementing a BM model and 20 genes show uniquely a gene expression-level shift in optimum when using an OU model (Figure S6).

**Figure 8:**
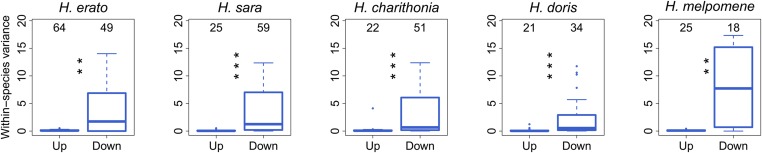
The gene expression variances for all the genes showing a shift toward an up- and a downregulation are depicted as box plots for each species. Numbers above the box plots show the total number of genes identified with a BM and an OU model. Wilcoxon test: * *P* < 0.05, ** *P* < 0.01, and *** *P* < 0.001. BM, Brownian motion; OU, Ornstein–Uhlenbeck.

Next, we assessed gene expression variance of all the genes identified as having a branch-specific shift in gene expression through BM and OU models. When we plotted the distribution of the gene expression variance, we found that upregulated genes have a significantly lower variance when compared to genes with a gene expression shift toward downregulation (Figure 8).

## Discussion

### Gene expression evolution through genetic drift

Our study on the evolutionary forces acting on gene expression in combined eye and brain tissue of *Heliconius* butterflies reveals that drift is the dominant evolutionary force driving gene expression divergence (81% of the transcriptome). According to neutral expectations, phenotypic changes are expected to accumulate as a function of time, by drift and mutation alone (Lande 1976). Following this rationale, the change of transcriptomic levels through drift should reflect the divergence history of the taxa of interest. From our BM analysis, we show that in most of the gene expression levels in combined eye and brain, a phylogenetic signal can be recovered (Figure 3). Nevertheless, we have to keep in mind that a phylogenetic signal can also be recovered even if other evolutionary forces are acting on the transcriptome, such as stabilizing selection or directional selection (Harmon *et al.* 2010). There are other evolutionary scenarios, beside drift, which resemble a random walk as modeled by BM. For example, directional selection that varies in strength and direction in a random fashion from one generation to the next can also be described as having a BM behavior. Similarly, for strong stabilizing selection, when the trait’s optimum changes randomly, It can also be described by BM. For example, in a study investigating pulsed evolution in vertebrates, the authors included BM to model incremental phenotypic change, where the trait of interest followed a wandering optimum (Landis and Schraiber 2017). Thus, drift, randomly varying selection, and varying stabilizing selection can be modeled by BM.

Evolutionary rates of gene expression, which have been investigated at both population and species level, show that the proportion of the type of evolutionary force acting on transcriptomic levels is not constant across taxa (Whitehead and Crawford 2006a; Landis and Schraiber 2017; Nourmohammad *et al.* 2017; Stern and Crandall 2018). For example, when examining the evolutionary forces acting on gene expression levels in several fish populations, the authors reported that the dominant force driving expression changes was genetic drift (Whitehead and Crawford 2006b). Comparably, in studies concerning primates, genetic drift was the main force driving gene expression evolution (Khaitovich *et al.* 2005b; Chaix *et al.* 2008). The proportion of gene expression levels evolving by drift depends on the strength of natural selection acting on the interrogated transcriptome. For example, in a comparison between different organ types in mammals, gonad gene expression showed the lowest phylogenetic signal when compared to other organs like cerebellum or heart (Brawand *et al.* 2011). In *Heliconius* butterflies, other organs would need to be tested to get a more global understanding of how gene expression is evolving in the whole organism.

**Figure 9:**
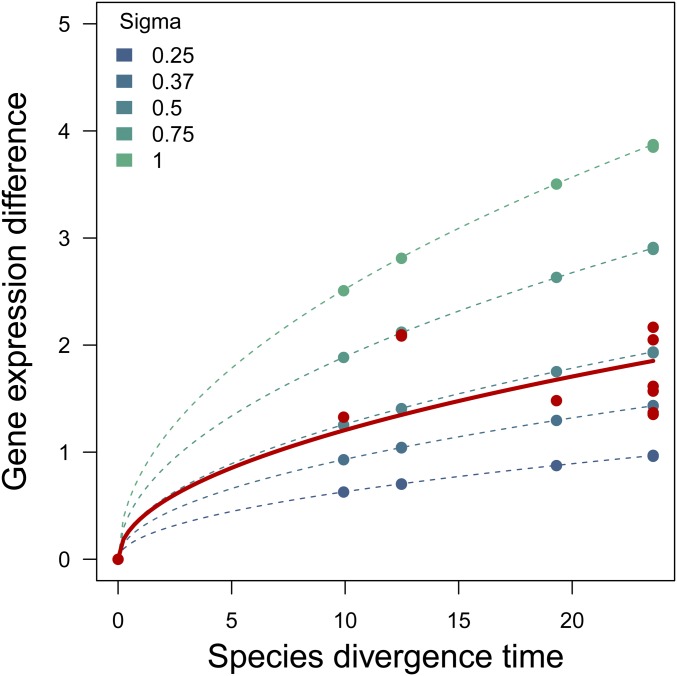
Between-species gene expression divergence plotted as a function of divergence time according to the *Heliconius* phylogeny. Red: σ^2^ from gene expression levels observed in *Heliconius*. Blue: simulated gene expression divergence under random drift with different values of σ.

We further explored our expression data by comparing the expected gene expression divergence under a BM model to the observed data. Consequently, we simulated expression levels for 10,000 genes along the known *Heliconius* phylogeny and computed the mean of the pairwise species differences. Similarly, we computed the mean pairwise differences of the observed gene expression data. Alternatively, we can also derive the expected divergence in gene expression levels between two species over time under BM. Both species evolve under random drift and, thus, their gene expression values are normally distributed with variance σ^2^ × *T*, where *T* is the time since the most recent common ancestor of the two species. Therefore, the difference in gene expression levels between the two species is normally distributed with variance 2 × σ^2^ × *T*. Since we are only interested in the absolute value of the gene expression difference, we use a truncated normal distribution instead. From this truncated normal distribution with mean zero and variance 2 × σ^2^ × *T*, we compute the expected gene expression difference through time (Figure 9). For the empirical data, we estimate σ^2^ using a sum of squares approach. We find that our observed gene expression data have a close fit to the simulated data (Figure 9).

### Gene expression evolution through stabilizing selection

Studies done in *Drosophila* and mammals have shown that stabilizing selection is the main evolutionary force driving gene expression evolution (Rifkin *et al.* 2003; Lemos *et al.* 2005; Rohlfs and Nielsen 2015). In contrast to these studies, in *Heliconius*, we discovered that only 3% of gene expression levels are either highly conserved (Figure 4) or are evolving through stabilizing selection (Figure 5). Factors such as tissue type, gene functionality turnover, or epistatic levels have the potential to influence the degree of stabilizing selection acting on the transcriptome (Larracuente *et al.* 2008; Kalinka *et al.* 2010; Romero *et al.* 2012). Additionally, in groups that have experienced an adaptive radiation, such as *Heliconius* butterflies (Kozak *et al.* 2015), and have thus recently experienced an elevated rate of trait evolution, directional selection might be more recurrent than stabilizing selection. Factors such as the evolutionary history, the topology of the phylogenetic tree (*e.g.*, the depth of the phylogeny), and the type of continuous trait being studied will determine if a model describing drift or stabilizing selection describes the data best (Fay and Wittkopp 2008).

OU models are suitable models to study the force of stabilizing selection acting on a phenotype since the α parameter simulates the strength of selection keeping a trait close to an optimum (Beaulieu *et al.* 2012), as several studies exemplify (Kalinka *et al.* 2010; Brawand *et al.* 2011; Stern and Crandall 2018). When we applied an OU model to identify stabilizing selection on gene expression, we detected parameter estimation biases as shown by our simulation study (Figure 6). For small phylogenies, accurate parameter estimation is challenging since statistical power is weak with small sample sizes (Rohlfs *et al.* 2014), and parameters like α and σ^2^ tend to be overestimated (Beaulieu *et al.* 2012). Specifically, it is very challenging with small phylogenies to distinguish between conserved gene expression levels due to low values of drift (*i.e.*, no change) *vs.* high values of directional selection (*i.e.*, drift is removed due to selection) (Appendix II). Therefore, we propose that for small phylogenies, testing for σ^2^ = 0 under a BM framework and assessing for significance by applying Monte Carlo simulations is a better approach to uncover stabilizing selection. From our estimates on the rate of gene expression evolution (σ^2^ ranging from ∼0 to 9) and using Monte Carlos simulations to test for a σ^2^ significantly different from zero, we show that for 88% of the data we have a false discovery rate ≤ 5%. Thus, the likelihood that our Monte Carlo simulation approach for assessing conserved genes is reporting a false positive is very low for larger values of the rate of evolution (Appendix II).

When using this approach, we identified 83 genes with conserved gene expression levels across species. These genes might be involved in maintaining conserved processes that are essential for the function of eye and brain tissue in *Heliconius*. For example, from the top 10 genes with the most conserved gene expression levels, we found the transcription factor *bobby sox* (*bbx*) (Group_674, Appendix I). BBX belongs to the high-mobility box domain superfamily, which is involved in transcription, replication, and chromatin remodeling (Chintapalli *et al.* 2007). BBX has also been found to have orthologs in flies, humans, and mice (Nitta *et al.* 2015), suggesting high essentiality of *bbx* expression. Another highly conserved orthocluster (Group_977, Appendix I) was annotated as *glaikit* (Chintapalli *et al.* 2007), which is known to be essential for the formation of epithelial polarity and nervous system development (Dunlop *et al.* 2004).

**Figure 10:**
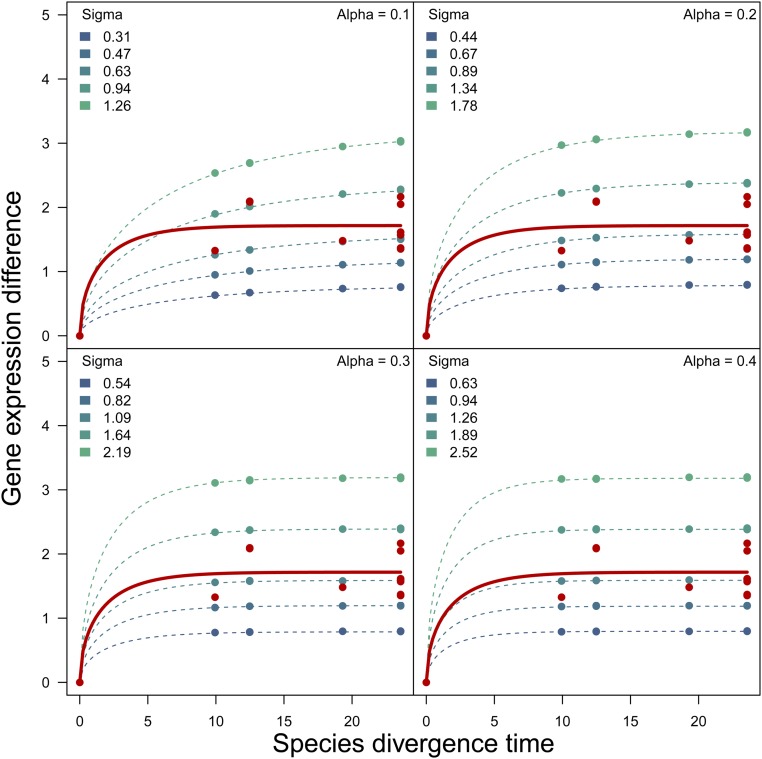
Between-species gene expression divergence plotted as a function of divergence time according to the *Heliconius* phylogeny. Red: σ^2^ from gene expression levels observed in *Heliconius*. Blue: simulated gene expression divergence under different values of σ. Each panel shows estimates for a different value of α.

To complement our BM approach, we further explored our data by simulating 10,000 genes under an OU model under a range of σ^2^ and α values, and computed the mean differences between pairs of species. We observed a reasonably good fit to our data (Figure 10), but it is worth pointing out that a steeper change in gene expression divergence is observed between closely related species when compared to the calculated expected divergence. This suggests that different evolutionary scenarios might explain the data better at different depths of the phylogeny, but could also be a characteristic signal of our data set. Consequently, adding more species, including those that are closely related, could not only improve OU parameter estimation but could also help disentangle the evolutionary forces acting on gene expression divergence, particularly between closely related species. Additional to the implementation of BM and OU models to individual genes (as we have done in this study), investigating how whole-gene networks or groups of coexpressed genes are evolving following BM/OU models along a phylogeny will increase the power to detect deviations to neutral expectation in small phylogenies (Schraiber *et al.* 2013). This approach was implemented in yeast where genes belonging to the same gene pathway were jointly analyzed, resulting in the identification of pathways with constrained and accelerated gene expression evolution, even when using small phylogenies. Such an approach could be explored with our data set once we have identified the genes that are evolving together because they form part of the same pathway.

### Gene expression evolution through genetic directional selection

To reveal branch-specific shifts in gene expression levels, we applied BM and OU models allowing for branch-specific shifts at the tips of the phylogeny. Using this approach, we found that 16% of the genes show a branch-specific shift, toward either up- or downregulation, with increased expression levels showing lower variance than expected (Figure 8, Figure S5, and Appendix II for false discovery rate analysis). The direction of a gene expression shift might be influenced by its mode of regulation. For example, in yeast, it was found that regulatory mutations affecting *trans*-regulatory factors were more likely to cause an increase in gene expression. Conversely, mutations in *cis*-regulatory elements were found to be skewed toward a decrease in expression (Metzger *et al.* 2016). On the other hand, it has been observed that mutations affecting gene expression are more prone to cause a downregulation as opposed to those mutations causing an upregulation, which tend to be less frequent and to cause bigger expression changes (Khaitovich *et al.* 2005b). Because random mutations increase entropy, there is a higher chance that a mutation in a regulatory region will decrease or disrupt the binding site of a transcription factor causing downregulation (Chaix *et al.* 2008). Investigating the mutation dynamics affecting gene expression variation in *Heliconius* will help us understand how mutational variance is linked to changes in gene expression (Hodgins-Davis *et al.* 2015).

Overall, if directional selection is causing a branch-specific shift in gene expression one would expect to see low within-species variance, whereas if the shift is caused by a relaxation of purifying selection or balancing selection, greater within-species variance would be expected. When we looked at the degree of variability between genes showing a shift toward a higher or a lower expression level, we observed that downregulated genes have significantly higher variance than genes showing upregulation (Figure 8). From this observation, we hypothesize that relaxation of purifying selection might be driving the shifts causing downregulation on gene expression, a pattern that could eventually lead to a loss of expression. However, balancing selection or experimental noise could also lead to elevated within-species variance. Because of the cost of gene expression, it is expected that only those genes that are essential and have fitness effects will continue to be expressed, whereas genes that are not will eventually stop being transcribed (Stern and Crandall 2018). However, a shift toward downregulation does not always have to be a consequence of relaxed purifying selection. For example, in the orthocluster with identifier Group_449_clean_0, a sevenfold lower expression shift was detected in the branch leading to *H. doris* (Figure S7) and significantly less variance than was expected transcriptome-wide (Fisher’s exact test, *P*-value < 0.001). Directional selection favoring downregulation of gene expression can occur in a scenario where fine-tuning of expression levels is necessary for optimal cell or tissue function (Cayirlioglu *et al.* 2008; Catalán *et al.* 2016).

On the other hand, genes showing a branch-specific shift toward upregulation have significantly lower variances when compared to expression level shifts toward downregulation (Figure 8). This observed pattern could be a result of directional selection acting on gene expression levels leading to a reduction in the variation observed in gene expression. It is possible that to achieve an increase in gene expression levels, the selective forces leading to upregulation would have to be sufficiently strong to result in a greater investment of energy allocated to transcription costs (Wagner 2005; Lang *et al.* 2009). Some of the genes having the most extreme branch shifts in expression, either toward a higher or a lower expression level, are involved in enzymatic activity (Appendix I). Enzymes support biochemical and physiological processes, helping the optimization of tissue function (Wagner and Altenberg 1996; Feller and Gerday 1997). Thus, optimal enzymatic activity might be a key factor for species-specific brain and eye function, which in turn might be optimized for the species-specific life history and ecological niche. An approach to further test for positive selection acting on the genes showing a branch shift would be to take a population genetic approach and identify selective sweeps (Fraser *et al.* 2010; Catalán *et al.* 2012, 2016). This approach would provide a second line of evidence for positive selection acting on the transcriptome but will require demographic studies for each species (Kim and Stephan 2002).

A factor possibly influencing the proportion of transcriptome levels found to be evolving through drift, or stabilizing or directional selection is the methodology used for orthology assessment. In our analysis of gene expression variation, we assessed variation in orthoclusters where an orthologous hit was found for each of our five *Heliconius* species. Additionally, because *de novo* transcriptome assemblies are prone to form chimeric transcripts, we used strict filtering criteria when assessing for orthology (see *Materials and Methods*). Genes with fast-evolving protein rates—to the point that orthology assessment becomes challenging—might also show gene expression shifts, which would not be detected in our experimental design. For example, orthology assessment for genes showing sex-biased gene expression, which tend to have higher evolutionary rates than unbiased genes, might require an alternative method. In fact, from the orthoclusters that we identified in this study, only two included genes with sex-biased expression (Catalán *et al.* 2018).

With this work, we have generated a set of candidate genes that are putatively evolving through directional selection, and that have the potential to be involved in the processes of adaptation and speciation. To test the role of these genes in such processes, functional validation will be necessary to gain deeper insight in the evolutionary consequences of gene expression shifts. Techniques like *in situ* hybridization, RNA interference, and clustered regularly interspaced short palindromic repeats/Cas9 are tools that can be used to shed light on the functionality of these genes. Particularly interesting could be those genes whose gene expression levels have shifted to the degree of showing absence of expression (Figure S8). The evolution of gain and loss of gene expression across a phylogeny requires a suitable theoretical framework that should be explored carefully, since such events have the potential to cause big phenotypic shifts.
